# The Role of Psychological Readiness in Return to Sport Assessment After Anterior Cruciate Ligament Reconstruction

**DOI:** 10.1177/0363546521991924

**Published:** 2021-03-03

**Authors:** Anne Gro Heyn Faleide, Liv Heide Magnussen, Torbjørn Strand, Bård Erik Bogen, Rolf Moe-Nilssen, Ingunn Fleten Mo, Willemijn Vervaat, Eivind Inderhaug

**Affiliations:** † Haraldsplass Deaconess Hospital, Bergen, Norway; ‡ University of Bergen, Bergen, Norway; § Western Norway University of Applied Sciences, Bergen, Norway; ‖ Haukeland University Hospital, Bergen, Norway; Investigation performed at Haraldsplass Deaconess Hospital, Bergen, Norway

**Keywords:** anterior cruciate ligament (ACL), return to sports, ACL reinjury, psychological aspects of sport

## Abstract

**Background:**

Knowledge about the predictive value of return to sport (RTS) test batteries applied after anterior cruciate ligament reconstruction (ACLR) is limited. Adding assessment of psychological readiness has been recommended, but knowledge of how this affects the predictive ability of test batteries is lacking.

**Purpose:**

To examine the predictive ability of a RTS test battery on return to preinjury level of sport and reinjury when evaluation of psychological readiness was incorporated.

**Study Design:**

Cohort study; Level of evidence, 2.

**Methods:**

A total of 129 patients were recruited 9 months after ACLR. Inclusion criteria were age ≥16 years and engagement in sports before injury. Patients with concomitant ligamentous surgery or ACL revision surgery were excluded. Baseline testing included single-leg hop tests, isokinetic strength tests, the International Knee Documentation Committee (IKDC) Subjective Knee Form 2000, a custom-made RTS questionnaire, and the Anterior Cruciate Ligament-Return to Sport after Injury (ACL-RSI) scale. The RTS criteria were IKDC 2000 score ≥85% and ≥85% leg symmetry index on hop and strength test. At a 2-year follow-up evaluation, further knee surgery and reinjuries were registered and the RTS questionnaire was completed again. Regression analyses and receiver operating characteristic analyses were performed to study the predictive ability of the test battery.

**Results:**

Out of the 103 patients who completed the 2-year follow-up, 42% returned to their preinjury level of sport. ACL-RSI 9 months after surgery (odds ratio [OR], 1.03) and age (OR, 1.05) predicted RTS. An ACL-RSI score <47 indicated that a patient was at risk of not returning to sport (area under the curve 0.69; 95% CI, 0.58-0.79), with 85% sensitivity and 45% specificity. The functional tests did not predict RTS. Six patients sustained ACL reinjuries and 7 underwent surgery for other knee complaints/injuries after RTS testing. None of the 29 patients who passed all RTS criteria, and were therefore cleared for RTS, sustained a second knee injury.

**Conclusion:**

ACL-RSI and age were predictors of 2-year RTS, while functional tests were not informative. Another main finding was that none of the patients who passed the 85% RTS criteria sustained another knee injury.

The definition of success after anterior cruciate ligament reconstruction (ACLR) is a matter of ongoing debate.^2,29,33^ For many patients, the major concern is whether a safe return to sport (RTS), without incurring reinjuries, is possible. A common expectation is to return to the preinjury level of sport participation, often in demanding activities involving jumping, pivoting, and cutting.^2,3,14,21^ These goals seem difficult to reach, as recent reports suggest that only 65% of patients return to their preinjury level of sport and only 55% to competitive sports.^3^ For those who return to cutting or pivoting sports, the risk of reinjury is high. Up to 30% suffer a second ACL injury, with the young, active population at greatest risk.^9,40,41,52^

RTS testing after ACLR has emerged to help assess patients’ readiness for the resumption of former activities. A range of test batteries with various criteria for RTS has been suggested.^2,7,40,53^ As there is little knowledge on the validity of these tests, we do not know which test—or combination of tests—can help us predict a timely and safe RTS.^2,10,28,40,49^ Establishing predictive validity is therefore a much-needed step in the further development of readiness test batteries.^2,10,45^

RTS is multifactorial, requiring both physical and psychosocial recovery after surgery.^5,10^ Physical functioning assessment has traditionally dominated RTS evaluation, but there is emerging evidence for incorporating psychological factors in these decisions.^2-4,6,25^ The Anterior Cruciate Ligament–Return to Sport after Injury (ACL-RSI) scale evaluates patients’ psychological readiness to RTS. Adding the scale in the RTS assessment is recommended,^2,34,51^ but little is known about how this affects the predictive validity of RTS test batteries.

Therefore, the aim of this study was to examine the predictive ability of a commonly used test battery on return to preinjury level of sport and reinjury when evaluation of psychological readiness was incorporated. The hypothesis was that a combination of physical function and psychological readiness would better predict success than physical function alone.

## Methods

### Patient Selection

**Figure 1.**
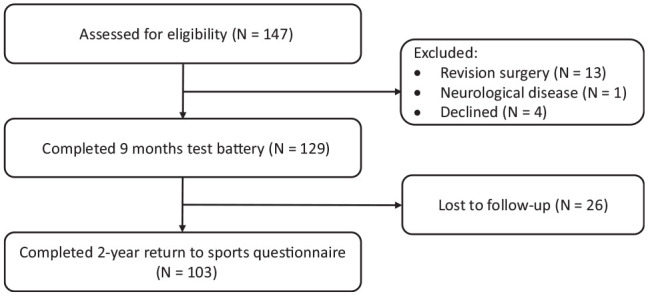
Flowchart of study participants.

From 2015 to 2018, patients in this cohort were prospectively recruited at the 9-month follow-up after ACLR at a local hospital’s orthopaedic clinic. Inclusion criteria were age ≥16 years at inclusion, fluency in Norwegian, and being engaged in physical activity or sports before injury. Exclusion criteria were concomitant ligamentous surgery or ACL revision surgery. Patients who declined functional testing, or had incomplete test battery results (ie, were unable to perform hop tests), were excluded from analyses. Of 147 patients screened for eligibility, 129 were enrolled in the study after exclusions (Figure 1). All patients gave their written, informed consent before inclusion. The study was approved by the regional committee for medical and health research ethics (ID No. 2016/1896). Patients in this cohort also participated in a validity study of the Norwegian language version of the ACL-RSI.^13^ All patients recruited to the validity study from the current clinic were screened for eligibility in the present study.

### Testing Procedure

Baseline testing of all patients was performed 9 months after ACLR. At this point, any early ACL reinjuries to the same, or contralateral, knee were registered. A custom-made RTS questionnaire was completed (Table 1). To enhance comparability with other studies, sports levels were also defined by the International Knee Documentation Committee (IKDC) as Level I sports, which include pivoting, hard cutting, and jumping movements (ie, soccer); Level II sports, which comprise lateral movements and sports with lesser pivoting (ie, alpine skiing); and Level III sports, which involve straight-ahead activities (cycling and running).^17,18,21^

**Table 1:** Sports and Activity Before and After ACLR^*a*^

Questions		Answer Options
1.	What was your main sport/activity before injury?	Soccer, team handball, basketball, etc.
2.	At what level did you perform your sport/activity before injury?	(1) Elite, (2) Medium to high competitive, (3) Low competitive, (4) Recreational
3.	What is your goal for return to sport/activity after surgery?	Type and level are specified as above
4.	At what level do you perform your main sport/activity now?	(1) Elite, (2) Medium to high competitive, (3) Low competitive, (4) Recreational
5.	If your goal was returning to another sport/activity: At what level do you perform that sport/activity now?	(1) Elite, (2) Medium to high competitive, (3) Low competitive, (4) Recreational

a English summary of content. ACLR, anterior cruciate ligament reconstruction.

### Measurements

The ACL-RSI scale was used to measure psychological readiness for RTS.^51^ The questionnaire comprises 12 questions covering key aspects of RTS: emotions related to returning (eg, fear and frustration), confidence in sports performance, and appraisal of reinjury risk.^51^ For example, a question about reinjury is “Are you fearful of reinjuring your knee by playing your sport?”^51^ Patients grade their answers from zero to 100 with 10-point increments. A total score is calculated as the average of the responses on each question, and higher scores indicate greater psychological readiness.^50,51^ The Norwegian version of the ACL-RSI is valid and reliable for patients after ACLR.^13^

The IKDC Subjective Knee Form 2000 was used to measure symptoms, function, and sports activity.^23^ The score ranges from zero (low function) to 100 (high function).^23^ The IKDC 2000 has adequate validity and reliability for patients with knee injuries.^11,23^

The single-leg hop test was used as a performance test to measure dynamic knee stability.^15^ It comprises 4 tasks: single hop for distance (in centimeters); triple hops for distance (in centimeters); triple crossover hops for distance (in centimeters); and 6-m timed hops (in seconds).^35,37^ The uninvolved leg was tested first. The results are presented as a mean Limb Symmetry Index (LSI%; the percentage difference in the performance between limbs) of the 4 tasks. A score of 100% means there is complete symmetry in the performance of the legs. Values <100 indicate a deficit in the involved leg.^40^ Hop tests are reliable and valid for patients after ACLR.^27,44^

Concentric knee extension strength was measured at 60 deg/s (5 repetitions) angular velocity using an isokinetic dynamometer testing system (Biodex System 3 Dynamometer; Biodex Medical Systems Inc). The uninvolved leg was tested first. Performance is reported as an LSI (%) in peak torque (PT) Newton meters (N·m). Isokinetic strength tests are reliable and valid outcome measures after ACLR.^47,49^

### RTS Criteria

The earliest point where patients were advised to return to pivoting sports was 9 months after surgery, as recommended by Grindem et al.^20^ The conventional test battery used for RTS clearance consisted of the IKDC 2000, single-leg hop tests, and concentric knee extension strength. The RTS criteria were IKDC 2000 score ≥85%, ≥85% LSI on hop test, and ≥85% LSI on isokinetic strength test (extension PT 60 deg/s). If a patient was returning to IKDC Level I or Level II sports at higher competitive levels, the criteria were adjusted to 90%. Patients who did not pass the criteria were advised against returning to Level I or II sports and were given the opportunity to return for repeat testing.

### Two-Year Follow-up Evaluation

Two years after surgery, the RTS questionnaire was used to acquire data on return to sport and level of participation. Meniscal and cartilage surgery (resection or repair), or additional surgery to knee ligaments, were registered between baseline and follow-up. Furthermore, details on any reinjuries were acquired based on telephone interviews and data from routine clinical follow-ups performed by experienced orthopedic surgeons. An ACL reinjury was defined as a graft rupture or contralateral ACL rupture confirmed by either (1) arthroscopy, (2) magnetic resonance imaging, or (3) anamnestic episodes of knee trauma followed by an increased objective instability compared with earlier controls (KT-1000 arthrometer [Medmetric] ≥5, Lachman test 2+ or pivot-shift test 2+).

### Surgical Technique and Postoperative Rehabilitation

The ACLR was performed arthroscopically by an anatomic technique using either the patellar tendon or hamstring tendon autograft from the ipsilateral knee. No brace was used and immediate weightbearing was allowed, supported by crutches for 2 to 4 weeks. For patients who underwent additional surgery (such as meniscal repair), progression of rehabilitation was adjusted according to restrictions. Before hospital discharge, all patients performed postoperative supervised exercises and received guidelines regarding exercise progression and advice on contacting a physical therapist for further guidance. If the knee was effusion-free and the patient had a satisfactory range of motion and muscular control, running was allowed after 12 weeks. Gradual sport-specific training was allowed 6 months after surgery (ie, participating in team warm-ups/training, but not playing football or handball).

### Statistical Analysis

IBM SPSS Statistics Version 24.0 software (IBM Corp) was used for analyses. For continuous variables, means ± SD are presented, and for categorical variables, absolute and relative frequencies are presented. Between-group comparisons were made by independent samples *t* tests, chi-square analyses, and Mann-Whitney U tests as appropriate. Logistic regression analyses were used to examine the predictive ability of questionnaires (ACL-RSI and IKDC 2000) and functional tests for return to preinjury sport level 2 years after surgery, with and without adjustments for age and sex. The variables were entered as continuous variables, not applying the 85% cutoffs. In addition, variables (age, sex, and time from injury to surgery) that could potentially affect RTS were examined separately in the logistic regression. To further examine the predictive ability of the complete test battery, stepwise backward multivariate logistic regression was performed. Results are presented as odds ratios (ORs), 95% CIs, and amount of explained variance (Nagelkerke *R*^2^). Variables with significant association with RTS in the final stepwise backward model were entered into a receiver operating characteristic (ROC) model to evaluate predictive ability. A separate ROC analysis was performed for the ACL-RSI. Results are presented as area under the ROC curve (AUC), sensitivity, and specificity. The explanatory variables were checked for multicollinearity using linear regression analysis. Tolerance values <0.1 indicate unwanted high correlations between variables.^39^

## Results

### Patient Characteristics

For information on patient characteristics, see Table 2. Of the patients, 60% received a bone–patellar tendon–bone autograft and 40% received a hamstring tendon autograft. Fifteen patients had a history of ACLR in the contralateral limb. Of 103 patients, 69% performed IKDC Level I sports before injury; 16%, Level II; and 15%, Level III. Most patients stated that they wanted to return to their preinjury sport/activity (87). Seven patients stated that they had returned to full sports participation before RTS testing at baseline. Forty-three patients declined functional testing or had incomplete results. The 14 patients who declined or interrupted testing because of knee pain or instability had lower ACL-RSI scores than patients who did not perform testing because of other reasons (ie, lack of time or Biodex out of order; ACL-RSI, 35 vs 54; *P* = .001).

**Table 2:** Baseline Patient Characteristics (n = 103)^*a*^

Age at surgery, y	28.7 ± 10
Male sex	55 (53)
Median time from injury to surgery, mo (IQR)^*b*^	8 (11)
**Concomitant surgery**	
Meniscal resection	18 (18)
Meniscal repair	25 (24)
Cartilage debridement	1 (1)
Microfracture^*c*^	1 (1)
**Preinjury level of activity/sport**	
Elite	5 (5)
Medium/high competitive	29 (28)
Low competitive	37 (36)
Recreational	32 (31)
**Four main activities/sports**	
Soccer	51 (50)
Handball	13 (13)
Alpine skiing	6 (6)
Cross-country/mountain running	6 (6)

a Data are reported as n (%) or mean ± SD unless otherwise indicated. IQR, interquartile range.

b Information missing in 5 patients (n = 98).

c This patient also had a meniscal repair.

### Baseline Results

Baseline testing was performed on average 10.4 ± 1.3 months after ACLR. For information on measurements, see Table 3. Twenty-nine patients passed the ≥85% RTS criteria in all 3 tests (hop test, strength test, and IKDC 2000). These patients were younger (26 vs 30 years; *P* = .037), had higher ACL-RSI (69 vs 51; *P* < .001), and IKDC 2000 (92 vs 77; *P* < .001) scores and performed better on the functional tests (hop test sum score, 100% vs 95%; LSI and isokinetic strength test, 96% vs 78% LSI; *P* < .001) than those who did not pass. More patients performing IKDC Level I sports before injury passed (*P* < .001).

**Table 3:** Baseline Results of Psychological Readiness, Self-Reported Knee Function, and Performance on Functional Tests (n = 103)^*a*^

	All Patients (n = 103)	Returners (n = 43)	Nonreturners (n = 60)	Mean Difference (95% CI)	*P* Value
**Subjective scores**					
ACL-RSI (0-100, high score best)	55.8 ± 22.4	63.5 ± 20.8	50.3 ± 22.0	−13.3 (–21.9 to –4.8)	.003
IKDC 2000 (0-100, high score best)	81.4 ± 11.4	83.6 ± 9.8	79.9 ± 12.2	−3.8 (–8.2 to 0.7)	.099
**Hop tests**					
Mean sum score, LSI %	96.1 ± 8.5	97.0 ± 8.6	95.5 ± 8.4	−1.6 (–4.9 to 1.8)	.363
**Isokinetic strength test**					
PT extension 60 deg/s, LSI %	83.3 ± (14.8)	85.0 ± 14.2	82.0 ± 15.2	−2.9 (–8.8 to 2.9)	.324

a Data are reported as mean ± SD unless otherwise indicated. ACL-RSI, Anterior Cruciate Ligament-Return to Sport after Injury scale; IKDC 2000, International Knee Documentation Committee Subjective Knee Form 2000; LSI, limb symmetry index; PT, peak torque.

### New Injuries and Repeat Surgery at Follow-up

The final follow-up evaluation was undertaken at mean 25.5 ± 2.9 months after surgery. Six patients had sustained graft reinjuries (1 before RTS testing, 5 after) and 1 patient sustained a contralateral ACL injury between the baseline RTS testing and follow-up (5.8% reinjury rate). Three of those with an ACL reinjury returned to preinjury level sports although they had sustained graft failure. Seven patients underwent surgery for other knee complaints/injuries from RTS testing until follow-up evaluation: 4 patients had meniscal resections, 1 had a meniscal repair, 1 had cartilage resection, and 1 underwent a microfracture procedure. The total reinjury rate after RTS (combining ACL reinjuries and additional injuries) was 13.6%.

None of the 29 patients who passed the 85% RTS criteria were reinjured or underwent additional surgery after RTS testing compared with 13 reinjuries in the group who did not pass (*P* = .037). Fourteen (48%) of those who passed had returned to preinjury level sports compared with 29 (39%) of the 74 who did not pass (*P* > .05). Because of the low number of reinjuries, further analyses of predictive ability on new injuries were not feasible.

### RTS at Follow-up

A total of 43 (42%) patients had returned to their preinjury level of sport 2 years after surgery. Returners were older (mean age, 31 vs 27 years; *P* = .035) and had higher 9-month ACL-RSI scores (64 vs 50; *P* = .003) than nonreturners (Table 3). More patients performing at the recreational level returned to their preinjury level (*P* = .026). Patients participating at a recreational level were older than patients at competitive levels (mean age, 37 vs 25 years; *P* < .001)

### Predictive Ability on RTS

In the logistic regression, age, ACL-RSI, and IKDC 2000 had a significant association with returning to preinjury level of sport (Table 4). In the stepwise backward regression, the IKDC 2000 no longer displayed a significant effect: age and ACL-RSI were the only variables predicting RTS, with ORs of 1.05 (*P* = .037) and 1.03 (*P* = .005), respectively (Table 4). Of the variance in RTS, 17% could be explained by this model. For each 1-point increase in ACL-RSI score, the likelihood for returning increased by 3%. Tolerance values ranged from 0.55 to 0.88, indicating absence of multicollinearity. Results on backward regression did not change when patients with previous contralateral ACL injury were removed from analyses: age (OR, 1.06; 95% CI, 1.01-1.11; *P* = .022) and ACL-RSI (OR, 1.03; 95% CI, 1.01-1.06; *P* = .004) were still the only variables left in the final model.

**Table 4:** Unadjusted and Adjusted Binary Logistic Regression Predicting Likelihood of Returning to Preinjury Sport (n = 103)^*a*^

	OR	95% CI	*P* Value	*R* ^2^
**Separate logistic regression**				
**Age at surgery**	**1.05**	**1.00-1.09**	**.030**	**0.06**
Sex	0.61	0.28-1.36	.225	0.02
Time from injury to surgery^*b*^	0.75	0.99-1.02	.749	0
**ACL-RSI**	**1.03**	**1.01-1.05**	**.004**	**0.12**
**ACL-RSI adjusted**^*c*^	**1.03**	**1.01-1.05**	**.006**	**0.17**
IKDC 2000	1.03	0.99-1.07	.102	0.04
**IKDC 2000 adjusted**^*c*^	**1.04**	**1.09-1.09**	**.049**	**0.12**
Hop test, LSI%	1.02	0.97-1.07	.362	0.01
Hop test, LSI% adjusted^*c*^	1.02	0.97-1.07	.425	0.08
Isokinetic extension strength, PT 60 deg/s, LSI%	1.01	0.99-1.04	.322	0.01
Isokinetic extension strength, PT 60 deg/s, LSI% adjusted^*c*^	1.02	1.00-1.10	.138	0.10
**Stepwise backward regression, final model**				**0.17**
**Age**	**1.05**	**1.00-1.10**	**.037**	
**ACL-RSI**	**1.03**	**1.01- 1.05**	**.005**	

a Boldface indicated statistical significance. ACL-RSI, Anterior Cruciate Ligament-Return to Sport after Injury scale; IKDC 2000, International Knee Documentation Committee Subjective Knee Form 2000; LSI, limb symmetry index; OR, odds ratio; PT, peak torque.

b Information missing for 5 patients (n = 98).

c Adjusted for age and sex.

**Figure 2.**
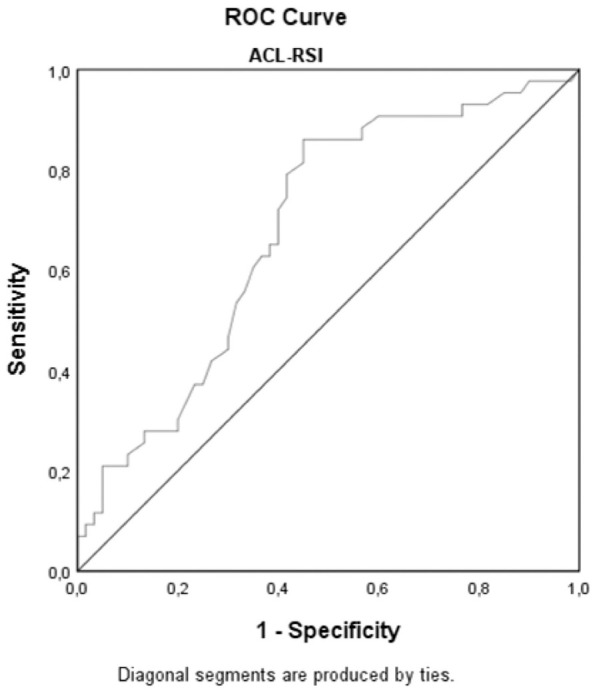
Receiver operating characteristic (ROC) curve for Anterior Cruciate Ligament-Return to Sport after Injury scale (ACL-RSI) for predicting return to preinjury level of sport.

**Figure 3.**
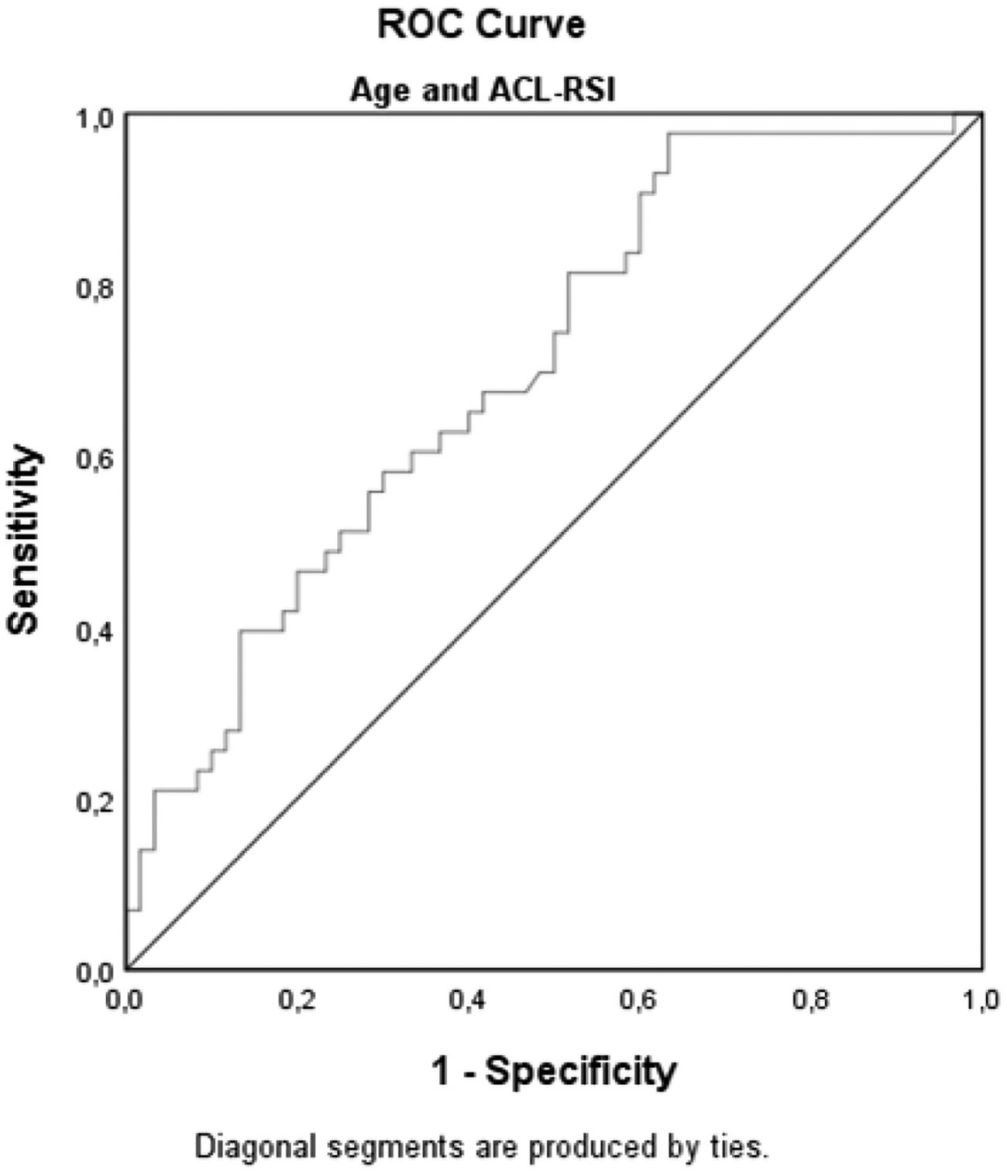
Receiver operating characteristic (ROC) curve for Anterior Cruciate Ligament-Return to Sport after Injury scale (ACL-RSI) and age for predicting return to preinjury level of sport.

For the ACL-RSI, the AUC was 0.69 (95% CI, 0.58-0.79; *P* = .002), with 85% sensitivity and 45% specificity at an ACL-RSI score of 47 (Figure 2). When ACL-RSI and age were combined in an ROC analysis, the AUC was 0.70 (95% CI, 0.60-0.80, *P* < .001), with a sensitivity of 98% and a specificity of 63% (Figure 3).

## Discussion

In the current study, age and psychological readiness displayed a predictive ability for return to preinjury level of sports, while conventional RTS tests did not. Of the patients, 42% returned to their preinjury level within 2 years after surgery. Those who returned were older and had better self-reported function and higher psychological readiness 9 months after surgery. The ACL reinjury rate was 5.8%. None of the patients who passed the ≥85% RTS criteria test battery sustained a second knee injury.

Few studies have examined the predictive ability of ACL-RSI for RTS in prospective cohorts. In the current study, patients’ ACL-RSI scores 9 months after surgery had a small, but significant, predictive ability on 2-year RTS. Similar findings are reported from cohorts comparable with the current cohort. Ardern et al^4^ found preoperative and 4-month postoperative scores to be predictive of return to preinjury level at 1 year after surgery. Sadeqi et al^46^ reported a greater predictive ability when regression analysis was performed with ACL-RSI as a binary outcome (cutoff, 60 points). The explained variance in the current study was low, but the ACL-RSI was developed to cover only psychological readiness.^51^ Mental factors such as recovery expectations and motivation may also influence the rehabilitation process.^4,6,42^ Further, factors related to surgery (ie, tunnel positioning) and rehabilitation (ie, different protocols) are also important for RTS.^4,8,12,22,48^ In this sense, the ability of the ACL-RSI to explain 12% of the variance in RTS outcomes alone can be considered a fairly good result.

Fair to good predictive ability is reported for ACL-RSI scores at 4 to 6 months’ follow-up with varying cutoffs (51.3-65.0), AUC values (0.77-0.80), and ranges of sensitivity (57%-97%) and specificity (63%-84%).^4,32,46,50^ In the present cohort, patients with ACL-RSI scores <47 were at risk of not returning to their preinjury level of participation, with a sensitivity of 85% and a specificity of 45% indicating a fair predictive ability. Knowledge on cutoff values will enable clinicians to identify patients in need of treatment strategies targeting unfavorable psychological responses.^51^ Hopefully, these strategies will contribute to improving patients’ overall readiness to resume sports, but more research is needed to clarify what the strategies should comprise.^4,51^ The relatively high sensitivity and the lower specificity means that the ACL-RSI is better at identifying patients who will struggle to resume sports than identifying those who will return (many false-positives). As the main focus for clinicians is to identify patients needing extra assistance in returning to sports, the high sensitivity is of great importance.

In the current study, older age was a predictor of return to preinjury level, even though it added only a small amount of explained variance in the final regression model (5%). This contrasts with other reports where younger age favored returning.^4,26,55^ The relatively high proportion of patients performing recreational-level sports in the present study can explain this finding. More patients performing recreational-level sports returned to their preinjury level, and patients in this group were significantly older; hence, more of these “older” patients returned.

Symmetrical single-leg hop performance has been associated with successful return to preinjury level of sport, and 6-month postoperative hop tests are reported to predict short- and long-term RTS, with up to 45% explained variance.^3,32,34^ These results differ from the current study, where no predictive ability was found for hop tests. Differences in patient populations can be a reason for the discrepancies, as comparative studies include larger proportions of patients performing pivoting sports, with fewer concomitant injuries at surgery.^32,34^ Isokinetic quadriceps strength, another common indicator for RTS readiness, also did not have an effect on sport resumption in the current study. Others have reported weak to no association between quadriceps strength and RTS.^12,32,36,55^ These results on functional tests are surprising but may emphasize that the controlled setting of isokinetic testing and hop tests represents different challenges than the unpredictability of sports participation. Including other aspects of function through movement quality analysis, open skill tasks, reactive agility tests, and sport-specific tests could potentially lead to functional tests being predictive of RTS.^2,16,40^

The relationship between self-reported knee function and RTS is unclear.^12^ Indications of a relationship between higher IKDC scores and return to preinjury level of sport have been reported.^3-5,26,55^ This was also found in the current study, but the effect disappeared as other factors were added to the regression analysis. An explanation for the lack of association between knee function and RTS may be that physical and psychological readiness to RTS do not always coincide.^4,12,25,43^ The relationship between psychological readiness and isokinetic strength and hop test LSIs has been investigated and little to no relationship seems to exist.^5,13,38^ This indicates that physical and psychological recovery are distinct and different constructs and both should be addressed in rehabilitation.^38^

Test batteries must be informative regarding risk of reinjury. An interesting observation in the current study was that none of the patients passing the 85% criteria were reinjured or underwent additional surgery. Similar findings were reported by Grindem et al,^20^ as only 1 out of 18 patients passing their RTS criteria suffered a new knee injury compared with 21 new injuries in the 55 nonpassers. Meeting the criteria on these conventional RTS tests was associated with a 92% lower reinjury rate.^19,20^ Another study found nonpassers of a comprehensive test battery to be 4 times more likely to sustain a graft rupture.^24^ Neither of these studies included psychological readiness evaluation, but 2 other studies have reported a higher risk for a second ACL injury in young patients with low ACL-RSI scores.^30,31^

Strengths of the present study include the prospective evaluation of both physical and psychological readiness to RTS in a population representative of many hospital and outpatient clinics. The current cohort was recruited from a public hospital and represents patients performing a broad spectrum of sports; many participated at a lower competitive level or a recreational level. Patients were given a standardized rehabilitation protocol and were followed by local physical therapists for the main part of the rehabilitation. The authors believe that information on the predictive ability of RTS assessments in a population such as this will provide useful information to many outpatient and orthopaedic clinics, as some of the previous research has been biased toward specialized clinics treating athletes.^19,24,54^ Further, to the authors’ knowledge, there are no other studies examining the predictive value of 9-month scores, and only 1 study has followed patients for up to 2 years.^46^ Testing at 9 months after surgery is relevant because this is the earliest time patients are advised to return to sports.^20^

The results of the present study may not be comparable with populations of elite athletes following strict protocols at specialized clinics. In accordance with other studies, the RTS criteria were set to 85% (90% for those returning to IKDC Level I/II sports at higher levels of competition).^10,20,25,49,56^ This is slightly lower than recommended by some and may limit comparison with other studies.^1,48^ We argue that knowledge on which cutoffs to use in different populations is still limited, especially in more heterogeneous patient groups. The independent variables were therefore analyzed as continuous data, not applying cutoffs. A further limitation may be the lack of movement quality assessment, as this has previously been found to predict RTS.^55^ Also, the use of LSIs may be debated. While some support their use,^19^ others have questioned it, as symmetrical performance alone will not provide information on whether patients have regained preinjury function.^15,26,48,55,56^ Interestingly, the results of the regression analyses did not change in the current study when patients with a previous history of contralateral ACL injury were removed from analyses. However, it cannot be ruled out that by evaluating movement quality or using different metrics (ie, absolute norm values or quadriceps strength/hop performance normalized to body weight), functional tests could have a predictive ability for RTS.

## Conclusion

This study highlights the importance of incorporating evaluation of psychological responses in RTS testing. Age and psychological readiness measured 9 months after surgery were found to be predictors of RTS 2 years after ACLR, while functional tests had no predictive value. None of the patients who passed the 85% cutoff in the current test battery sustained a new knee injury, which may indicate an association between functional tests and risk of reinjuries.
